# Sex hormone levels and sexual dysfunction in men after coronary artery bypass graft 

**Published:** 2012-09

**Authors:** Seyed Khalil Forouzannia, Mohammad Hassan Abdollahi, Seyedhossein Hekmatimoghaddam, Sadegh Ali Hassan Sayegh

**Affiliations:** 1 *Yazd Cardiovascular Research Center, Shahid Sadoughi University of Medical Sciences, Yazd, Iran.*; 2 *Department of Anesthesiology, School of Medicine, Shahid Sadoughi University of Medical Sciences, Yazd, Iran.*; 3 *Department of Laboratory Sciences, School of Paramedicine, Shahid Sadoughi University of Medical Sciences, Yazd, Iran. *

**Keywords:** *Coronary artery bypass*, *Sexual dysfunction*, *Sex hormones*, *Ejaculation*, *Impotence*

## Abstract

**Background: **Sexual dysfunction is one of the most common problems in men after coronary artery bypass graft** (**CABG). Etiology of sexual dysfunction in these patients may be psychological or organic due to hormonal changes.

**Objective:** The purpose was to evaluate the incidence and type of sexual dysfunction and changes in serum concentration of sex hormones in male patients undergoing on-pump CABG.

**Materials and Methods: **In this before and after study we enrolled 40 men aged less than 70 years who were candidate for on-pump CABG. Interviews were done by a physician before and 12 weeks after the operation in regard to the impact of surgery on their sexual activities. The serum levels of 6 sex hormones were also determined. The statistical tests used for data analysis included analysis of variance, McNemar's test and chi-square analysis**.**

**Results:** The mean±SD age of the patients was 51.27±7.86 years. Incidence of sexual dysfunction was 22.5% (9 cases) before and 47.7% (19 cases) after operation. Types of sexual dysfunction were premature ejaculation (5% before, 2.5% after), impotence (7.5% before, 12.5% after) and loss of libido (10% before, 32% after). The level of sex hormones were generally decreased after operation but it was statistically significant only for estrogen (p-value=0.02).

**Conclusion:** Sexual dysfunction and reduction in serum level of sex hormones are common in patients before on-pump CABG and mostly get worse after surgery. Complementary studies are suggested for prevention and treatment of sexual dysfunction.

## Introduction

Coronary artery disease (CAD) is one of the most common causes of morbidity and mortality in developed and developing countries. Coronary artery bypass graft** (**CABG) is the most common cardiac surgery around the world (1, 2, 3). One of the most important post-operative complications is sexual dysfunction after on-pump or off-pump CABG (2). Etiology of sexual dysfunction in these patients may be psychological or organic due to hormonal changes. Normal sexual function has common implications for emotional health and happiness, and sexual dysfunction may have negative impacts on psychologic life resulting to stress, depression, loneliness and decreased quality of social relationship (4). 

Sexual dysfunction may be classified into four main groups: loss of libido, erectile dysfunction (ED), ejaculatory insufficiency, and anorgasmic states. A sexually competent male must have desire for his sex partner (libido), divert blood from the iliac artery into the corpora cavernosa to achieve penile tumescence and rigidity (erection) adequate for penetration, then discharge sperm and prostatic and seminal vesicle fluid through the urethra (ejaculation), and experience a sense of pleasure (orgasm) (5, 6). 

ED, also known as impotence, is the inability to achieve and/or maintain a penile erection sufficient enough to participate in satisfactory sexual activity. Frequency of ED has significant relationship with age: 25% of 60-years-old men and 49% of 70-years-old men have ED (2). At least 75% of patients with heart disease experience one or more types of sexual dysfunction (7).

Androgenic hormones include dihydroepiandrostenedione (DHEA), DHEA sulfate, androstenedione, testosterone and dihydrotestosterone. The most important female sex hormones which also affect male sexuality are estrogen, LH, FSH and prolactin. Cardiopulmonary bypass (CPB) for on-pump CABG can affect serum levels of androgenic hormones, especially testosterone (8).

Because of the high frequency of sexuality issues in our patients, this study was designed to evaluate circulating sex hormone levels and the frequency and types of sexual dysfunction in men after on-pump CABG.

## Materials and methods

Forty married male patients that were candidate for elective on-pump CABG in Afshar Hospital, Yazd, Iran, in 2009 were enrolled in this before and after study which was approved by the ethics committee in Shahid Sadoughi University of Medical Sciences, Yazd, Iran. An informed consent form was signed by the participants. Surgical team was the same in all patients. Exclusion criteria included patients with history of drug consumption for sexual dysfunction before surgery, age higher than 70 years, consumption of any drug which potentially affects sexual function during the study, and patients candidate for off-pump CABG.

A tested questionnaire (an abbreviated and modified IIEF, the International Index of Erectile Function questionnaire) for determination of incidence and type of dysfunctions was filled by a physician 2 days pre-operatively and also 12 weeks after operation, to be sure that near-normal daily life is resumed (the usual rehabilitation time needed after cardiac surgery is about 6 weeks). Every question was described by the physician to become sure that the patient fully understands it. The recorded variables included age, history of diabetes mellitus, hypertension, hyperlipidemia, addiction, serum levels of sexual hormones including LH, FSH, prolactin, estrogen, testosterone, and DHEA, and any type of sexual dysfunction before and after surgery.

Blood samples for determination of sex hormone levels were taken and sent to the hospital laboratory before and 12 weeks after operation. 

**Statistical analysis**

Routine ELISA kits were used for analysis of hormones. Data were analyzed in SPSS-16 software by using analysis of variance, chi-square and McNemar's test. Any p-value<0.05 was considered as significant. 

## Results

Forty patients candidate for on-pump CABG were enrolled in this study. The mean±SD age of the patients was 51.27±7.86 years (range 37-67 years). The frequency of pre-operation variables including diabetic mellitus, hyperlipidemia, hypertension, drug addiction and cigarette smoking were 35%, 60%, 37.5%, 10% and 15% respectively. The CPB±SD time was 44.89±12.68 minutes with range of 22-87 minutes. Left ventricle dysfunction was mild, moderate and severe in 20%, 65% and 15% of patients, respectively. 

The overall incidence of sexual dysfunction was 22.5% before and 47.5% at 12 weeks after operation, which is statistically significant (p<0.001). Preoperative sexual dysfunctions included premature ejaculation (5%), impotence (7.5%) and loss of libido (10%). Post-operative sexual dysfunctions were premature ejaculation (2.5%), impotence (12.5%) and loss of libido (32%). No patient stated more than one problem. Post-operatively, sex hormones levels decreased but it was statistically significant only for estradiol (p=0.028) (Table I). 

**Table I:** Pre-operative and post-operative serum levels of sex hormones

**Hormone**	**Before surgery (Mean± SD)**	**After surgery (Mean± SD)**	**p-value (paired T-test)**
FSH	4.48 ± 2.35	3.96 ± 2.26	0.100
LH	4.08 ± 2.5	3.65 ± 1.97	0.252
Prolactin	10.83 ± 5.51	9.64 ± 5.09	0.185
Estrogen	23.6 ± 13.25	18.13 ± 11.58	0.028
Testosterone	5.88 ± 3.23	5.08 ± 2.96	0.146
DHEA	156.1 ± 109.7	144.7 ± 71.1	0.51

## Discussion

Sexual dysfunction is one of the most common complications following on-pump CABG surgery and an important cause of reduction in quality of life among patients (1, 8, 9).

There are few studies about this dysfunction after CABG. The authors published the results of another study on different types of cardiac surgery in which the incidence of sexual dysfunction was 20.1% before, and 76.4% at 12 weeks after the operation. Different sexual dysfunctions found in that study were impotence, premature ejaculation, and decreased or loss of libido in 6.5%, 4.3% and 9.3%, respectively before operation, and 34.8%, 21.5% and 20.1%, respectively 12 weeks after the surgery (10). Some studies have reported that plasma level of testosterone in patients with CAD is lower than those without CAD (11). Since heart disease prevalence is higher in elderly and there is concomitantly reduced testosterone levels (12), administration of testosterone in elderly patients can improve sexual activity, decrease latency and provoke erection.

In evaluation of correlation between CAD and sexual dysfunction, age is a confounding variable (13). However, in this study we compared the incidence of sexual dysfunction before and after CABG in patients less than 70 years old.

We observed sexual dysfunction in 22.5% (9 cases) before operation and in 47.5% (19 cases) after operation, which is statistically significant (p=0.000). Cigarette smoking has been implicated in the pathophysiology of cardiovascular disease and as a risk factor for erectile dysfunction. In a study conducted by Chew *et al* it was reported that compared with never-smokers, former-smokers and ever-smokers have significantly higher erectile dysfunction (14). The high number of smoker patients in our study may explain high prevalence of post-CABG sexual dysfunction.

Chew *et al* carried out two other studies about cardiovascular mortality in men with erectile dysfunction, and concluded that the risk of cardiovascular mortality is greater in men with ED (15, 16). In our study, we observed high incidence of ED in patients with CAD undergoing on-pump CABG. Therefore, it may be suggested that patients with cardiac dysfunction plus erectile dysfunction are susceptible to more long term morbidity and mortality. In contrast to our research, in a study carried out by Maggio *et al*, estradiol was increased after surgery (17).

It should be noted that post-CABG plasma concentration of testosterone was lower than pre-operative concentration. Canbaz *et al* reported that level of testosterone was decreased significantly one day after CPB, but returned to preoperative levels seven days after the surgical procedure (8). Jackson *et al* indicated that total testosterone and free testosterone levels should be measured in all men with ED in accordance with contemporary guidelines in those who fail to respond to phosphodiesterase 5 (PDE5) inhibitors or have a chronic illness associated with low testosterone. For these patients testosterone replacement therapy may lead to symptomatic improvement and enhance the effectiveness of PDE5 inhibitors (18). We also suggest testosterone replacement therapy after cardiac surgery to improve SD.

In a study conducted by Trotter *et al,* pre-operative estradiol level was 29 pg/ml and post-operative level was 15 pg/ml, that is statistically significant, similar to our study. Pre-operative progesterone level was 0.13 ng/ml which increased to 0.9 ng/mL postoperatively (19). Verderber *et al* indicated that one of the most important recovery markers of patients after cardiac surgery is to be potentially capable of returning to social and sexual activity (20).

Coronary artery bypass grafting with cardiopulmonary bypass causes an acute stress response, acute and sudden changes in the circulation and hemostasis characterized by changes in the levels of sexual hormones (21). Furthermore, Heaton *et al* showed that CABG has significant negative effect on sexual activity. The most important cause of loss of libido, impotence and premature ejaculation in on-pump CABG may be acute changes of circulation and hemostasis (22). In a study conducted by Khan *et al* it was found that on-pump CABG had more long-term negative effects on quality of life compared to off-pump CABG, and the most important reason of it is extracorporeal circulation in on-pump procedure (23).

## Conclusion

In conclusion, this research showed that on-pump CABG can reduce sexual activities and sex hormone levels especially estradiol in men less than 70 years old. According to high incidence of sexual dysfunction and decrease in sexual hormone levels, we suggest additional studies, especially with long-term follow-up, to evaluate interventions for prevention and treatment of sexual dysfunction after heart surgeries.
